# Determination of Zygosity in Adult Chinese Twins Using the 450K Methylation Array versus Questionnaire Data

**DOI:** 10.1371/journal.pone.0123992

**Published:** 2015-04-30

**Authors:** Biqi Wang, Wenjing Gao, Canqing Yu, Weihua Cao, Jun Lv, Shengfeng Wang, Zengchang Pang, Liming Cong, Hua Wang, Xianping Wu, Liming Li

**Affiliations:** 1 Department of Epidemiology and Biostatistics, School of Public Health, Peking University, Beijing, 100191, China; 2 Qingdao Center for Diseases Control and Prevention, Qingdao, 266033, China; 3 Zhejiang Center for Disease Control and Prevention, Hangzhou, 310051, China; 4 Jiangsu Center for Disease Control and Prevention, Nanjing, 210009, China; 5 Sichuan Center for Disease Control and Prevention, Chengdu, 610041, China; MOE Key Laboratory of Environment and Health, School of Public Health, Tongji Medical College, Huazhong University of Science and Technology, CHINA

## Abstract

Previous studies have shown that both single nucleotide polymorphisms (SNPs) and questionnaires-based method can be used for twin zygosity determination, but few validation studies have been conducted using Chinese populations. In the current study, we recruited 192 same sex Chinese adult twin pairs to evaluate the validity of using genetic markers-based method and questionnaire-based method in zygosity determination. We considered the relatedness analysis based on more than 0.6 million SNPs genotyping as the golden standards for zygosity determination. After quality control, qualified twins were left for relatedness analysis based on identical by descent calculation. Then those same sex twin pairs were included in the zygosity questionnaire validation analysis. Logistic regression model was applied to assess the discriminant ability of age, sex and the three questions in zygosity determination. Leave one out cross-validation was used as a measurement of internal validation. The results of zygosity determination based on 65 SNPs in 450k methylation array were all consistent with genotyping. Age, gender, questions of appearance confused by strangers and previously perceived zygosity consisted of the most predictable model with a consistency rate of 0.8698, cross validation predictive error of 0.1347. For twin studies with genotyping and\or 450k methylation array, there would be no need to conduct other zygosity testing for the sake of costs consideration.

## Introduction

Zygosity determination, which was based on the description of genetic relationship between two individuals within a twin pair, is the foundation of all twin studies[1]. In terms of relatedness analysis across the genome, it is important to consider the issues that attribute to the quality and quantity of the DNA samples, as well as the types of markers. Two types of markers are currently used for zygosity determination: a set of multi-allelic polymorphic short tandem repeats (STRs) and numerous biallelic single nucleotide polymorphisms (SNPs)[2, 3]. Both markers markedly increase the precision of zygosity determination, which allows a level of misclassification close to zero [4–6].

Some studies showed that Infinium 450k methylation array is a powerful technique in terms of reagent costs, time of labor, sample throughput and coverage. It holds great promise for the better understanding of the epigenetic component in health and disease [7, 8]. 450k methylation array has been widely used in twin studies to unveil the epigenetic mechanism behind the metabolic diseases, cancers, pain sensitivity, hearing and aging [9–14]. The manufacturer suggests that there are 65 SNPs included on the 450k methylation array that can be used to generate a DNA "fingerprint" of their samples as an added level of quality control (S1 Table). By plotting the beta values from the 65 SNPs methylation profile in a scatter plot, investigators can check sample duplications [15]. Similar to the discovery of duplicate samples, 65 SNPs in the methylation array per se may be used to distinguish monozygotic (MZ) twins from dizygotic (DZ) twins without any other genetic information. The two aforementioned zygosity determination methods are all based on DNA testing. The expense and time-consuming nature of DNA testing make it infeasible in large-scale epidemiology studies. An alternative method, the questionnaire-based zygosity determination, has been widely implemented to diagnose MZ and DZ with the advantages of simplicity and accuracy. Zygosity questionnaire typically inquires two aspects of information: the degree of perceived resemblance by twins themselves and whether their appearance confused by others. The time to answer those questions is usually less than 5 minutes [16, 17]. The consistency rate of zygosity questionnaire varies from 0.90 to 0.98 among different studies [16–20]. Questionnaire based zygosity determination is commonly applied to describe the demographic distribution of twins and calculate the genetic and environmental contribution on phenotype.

We have two objectives in the current study: 1) to examine the value of 450k methylation array in zygosity determination; 2) to validate the zygosity questionnaire in Chinese adult twins.

## Materials and Methods

### Participants

The Chinese National Twin Registry (CNTR) is the first and also the largest population-based twin registry in China, where more than 30,000 twin pairs of all age and sex have been enrolled since 1999[21]. Based on the registry, a total of 480 adult twins were recruited from four provinces (Shandong, Zhejiang, Jiangsu and Sichuan) by the local general practitioners and centers for disease control and prevention during 2011–2013. The majority of twins were from rural areas. The participants responded to a questionnaire about zygosity, medical histories and other contact information. 2 ml peripheral blood samples were collected upon informed consent. Six twins were excluded because their twin siblings’ information was missing. Forty-five opposite-sex twin pairs were considered to be DZ twins and further excluded for zygosity determination. A hundred and ninety-two pairs (61 female-female twin pairs, 131 male-male pairs) were left after the exclusion. The age ranged from 18 to 81, with a mean of 44.8 years. The study protocol was approved by the ethical review of Peking University Biomedical Ethics Committee (IRB00001052-13022).

### Genotyping and Quality Control

A genome-wide genotyping scan among 192 pairs was carried out using Illumina HumanOmniZhongHua-8 BeadChip. High-quality genotyping was performed by laboratory specialized in Illumina SNP array genotyping following standard experimental procedures suggested by the manufacturer. A total of 894,956 SNPs were genotyped among 192 paired subjects. SNPs with minor allele frequency <0.05 (169,045 SNPs), Hardy-Weinberg equilibrium P <0.0001(23,170 SNPs), and SNPs call rate<97% (14,138SNPs) were excluded. Twins with a call rate less than 90% and their twin siblings were also ruled out for further zygosity analysis. Genome-wide heterozygosity of each individual was estimated to exclude cross contamination between samples. This test was performed using a subset of 155,588 SNPs pruned for linkage disequilibrium (r^2^ < 0.3). Two samples demonstrated signs of contamination (mean observed heterozygosity = 0.3404, standard deviation = 0.0052), those two samples and their twin siblings were excluded. Finally, 180 pairs of twins with 695,406 SNPs remained after the quality control filters.

### Genome-wide Methylation Profiling

All 192 pairs of DNA samples were assessed for integrity, quantity and purity by electrophoresis and NanoDrop measurements. Samples were randomly distributed into 96-well plates. Bisulfite-conversion of 800 ng of genomic DNA was performed using an EZ DNA methylation Kit (ZYMO research) according to the manufacturer’s recommendations. Briefly, samples were whole genome amplified followed by an enzymatic end-point fragmentation, precipitation, resuspension, hybridization and staining. Finally, the BeadChips were imaged using an Illumina iScan.

By using the GenomeStudio Software 2010 Methylation module, DNA methylation level was displayed as beta-values ranging from 0 to 1. Beta-value was defined as the algorism M/(M +U+100), where M and U represent the methylated and unmethylated signal intensities respectively. For the methylation quality control [22], we used the 65 SNPs to determine the genotype of the sample and compare it with genotype calls based on Zhonghua8 Beadchip data, to identify if there were some mixed-ups during methylation experiment. There were 4 samples and their twin siblings excluded because of the mixed-ups. Finally, 188 pairs were qualified for zygosity determination.

### Zygosity determination Questionnaire

Opposite sex twin pairs were considered to be DZ twins and were excluded in the questionnaire zygosity determination. We selected 192 same sex twin pairs to answer three questions about zygosity. The first one was about appearance similarity——“Could a stranger distinguish you and your sibling by yours current appearance?” with three options of Yes, No and Hard to say. The second one was about formal zygosity test——“Have you and your sibling taken a zygosity-diagnosing blood test?” and the answers were Yes, No and Do not know. The last one was about self previously perceived zygosity——“Which type of twins did you think that you and your sibling were?” with alternatives of MZ, DZ, and Do not know. The golden standard for zygosity determination among 192 twin pairs was based on the genotyping or methylation data.

### Statistical Analysis

We calculated the probabilities of twin and his sibling if their alleles are identical-by-descent (IBD). Usually, there were three probabilities for paired individuals to have zero, one or two pairs of IBD alleles. Those probabilities were denoted as Z_0_, Z_1_ and Z_2_ respectively. Almost all of the alleles were same for MZ twins, so their Z_2_ was 1, while Z_0_, Z_1_ were both 0. DZ twins were more like full siblings; their Z_0_, Z_1_ and Z_2_ were 0.25, 0.5 and 0.25. Then we calculated the proportion IBD which was defined as the formula Z_2_+0.5Z_1_. The proportion IBD of MZ twins was 1; while for DZ twins was 0.5. The IBD analysis was conducted by PLINK genome command [23]. A scatter plot with Z_0_, Z_1_ was mapped to discriminate MZ and DZ twins.

Zygosity determination by methylation array was conducted using two approaches. The first approach used IBD analysis as the genotyping data. The beta values of 65 SNPs were exported from GenomeStudio Methylation module. These beta values could be converted to SNPs genotypes because they would cluster, as much as they did in a standard genotyping theta graph [22, 24]. With the help of 450k methylation array annotation file, we could have the genotypes of those 65 SNPs. The second approach was based on the manufacturer’s guideline to identify the duplicates samples. Each pair was scatter plotted by the 65 SNPs beta values of one twin and his sibling. We calculated the intra pair correlation coefficients of the beta values and compared those coefficients between MZ and DZ twins by a box plot.

**Fig 1:**
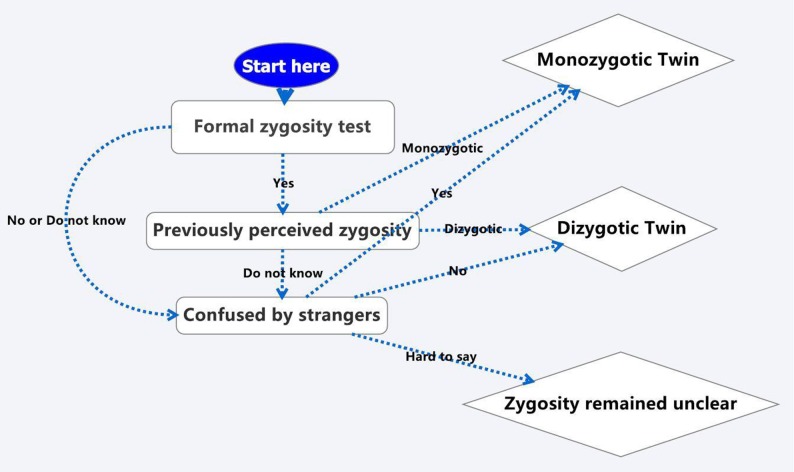
Complex strategy for zygosity determination based on the three question of formal zygosity test, previously perceived zygosity and confused by strangers. For example, if someone had “Yes” of Formal zygosity test, his zygosity would be the answer of question Previously perceived zygosity (PPZ) either “Monozygotic” or “Dizygotic” (but when he chose “Do not know” of question PPZ, his zygosity would be based on question Confused by strangers). On the contrary, when he chose “No” or “Do not know” in Formal zygosity test; his zygosity would be directly based on answers of Confused by strangers. In the question of Confused by strangers, “Yes” indicated to be identical twin, “No” referred to fraternal twin, “Hard to say” implied zygosity remained unclear.

We built six models to identify the best-predicted components in questionnaire for zygosity determination. Zygosity based on genotyping or methylation data was binary dependent variable (MZ or DZ) in each model. The 6 models were as follows: 1) Model 1 only included the question about self previously perceived zygosity (categorical variable as MZ, DZ or Do not know), noted as PPZ for short. 2) Model 2 only included the question about whether twins’ appearances confused by strangers (categorical variable as Yes, No or Hard to say), noted as CBS for short. 3) Model 3 was a complex strategy based on three zygosity question (see Fig 1, categorical variable as MZ, DZ or Do not know). 4) Model 4 included two independent variables, named PPZ and CBS respectively. 5) Model 5 was based on Model 4, plus an additional categorical variable of age group (less than 30 years old, 30 to 39, 40 to 49, 50 to 59 and 60 years and more). 6) Model 6 was based on Model 5 with an additional gender (male-male vs. female-female twins) variable.

Logistic regression model was applied to assess the discriminant ability of age, sex and three questions in zygosity determination with two-sided test at significant level 0.05. From logistic regression analysis, fitted probabilities were obtained from the data. Receiver operating characteristic (ROC) curves were plotted to determine the best cut-offs in terms of sensitivity and specificity, and the areas under the curve (AUC) was used as a measure of overall performance. Leave one out cross-validation (LOOCV) was used on all models as a measure of internal validation. All the analysis was conducted using *R packages*.

## Results

### Zygosity determination based on genotyping

**Fig 2:**
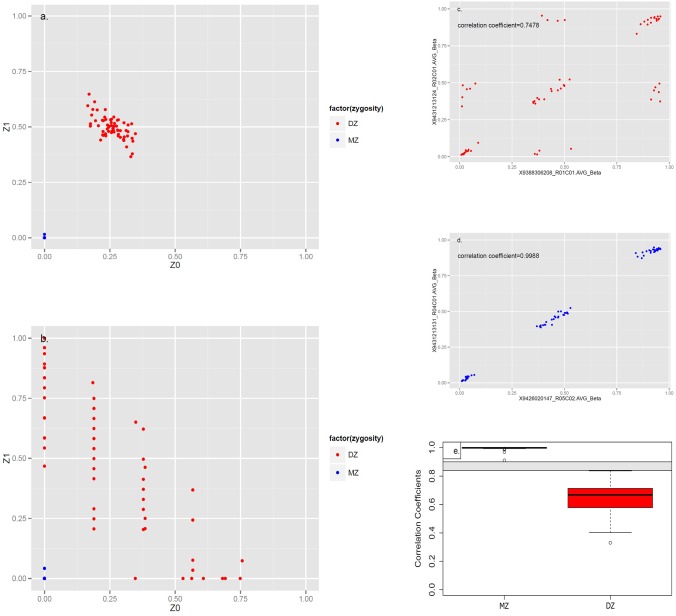
Relatedness Analysis of Discriminating Monozygotic\Dizygotic Twin Pairs by Genotyping and Methylation Array. (a) was the plot based on Genotyping Array Data, (b) was the plot based on Methylation Array Data. Z_0_, Z_1_ referred to identity-by-descent (IBD) probabilities: those for the individuals having zero or one pairs of IBD alleles. The Blue Dots indicated Monozygotic Twins (MZ), which Both Z_0_ and Z_1_ Approximately equaled to 0, while the Red Dots indicated Dizygotic Twins (DZ), which Z_0_ was close to 0.25 and Z_1_ was close to 0.5 by Genotyping Array Data. (c) was an scatter plot of methylation 65 SNPs beta values in a representative DZ pair with calculating the intra pair correlation coefficient 0.7478, (d) was an example of MZ pair with correlation coefficient 0.9988, (e) was the boxplot of methylation 65 SNPs correlation coefficient for MZ versus DZ, the grey area indicated possible cut-off points (correlation coefficient ranged 0.84–0.90) for discriminating MZ and DZ twins.

180 pairs with 695,406 SNPs were available for relatedness check analysis. The proportion of IBD was divided into two categories: one class with proportion of IBD varied from 0.9923 to 1 indicating MZ twins, while the other varied from 0.4094 to 0.5723 indicating DZ twins (S2 Table). The variance of IBD probabilities in DZ was greater than that in MZ twins (Fig 2A).

### Zygosity determination based on 450k methylation array

65 SNPs in the 450k methylation array were used to identify the zygosity among 188 pairs of twins. We conducted the same IBD analysis as using genotyping data to assess the discriminative ability for identifying MZ and DZ twins. The proportion of IBD with 65 SNPs had greater variation than the genotyping data. MZ twins had proportion of IBD either 0.9791 or 1, while DZ twins had a range of 0.2064 to 0.7662 (mean = 0.5035, standard deviation = 0.1355). The scatter plot of 65 SNPs was different from genotyping (Fig 2B). Although the blue dots, which were MZ twins, stayed in the lower left corner of the graph, they were more dispersedly distributed than genotyping data. The red dots, which were DZ twins, spread under the leading diagonal line. Nevertheless the greater variance has found with 65 SNPs, and there were no overlapped areas between MZ and DZ twins in the scatter plot.

By plotting the beta values of 65 SNPs in a scatter plot within a pair, we found representative samples (red dots) with an intra-pair correlation coefficient of 0.7478, scattered into seven possible spots (Fig 2C). That graph was an example of DZ twins. However, representative samples (blue dots) with an intra pair correlation coefficient of 0.9988, fell along the counter diagonal line (Fig 2D) that indicated to be MZ twins. Box plot was made to compare the intra pair correlation coefficients between MZ and DZ. The correlation coefficients varied from 0.9097 to 0.9953 in MZ, while it varied from 0.3304 to 0.8367 in DZ. The grey area in the box plot indicated possible cut-off points (ranged 0.84–0.90) for discriminating MZ and DZ twins (Fig 2E).

### Validation of the zygosity questionnaire

The characteristics of 192 same-sex twin pairs were presented in Table 1. There were no significant differences of age or gender between MZ and DZ twins. However, DZ twins were less likely to be confused by strangers, to have less formal zygosity test and regarded themselves as DZ or unknown zygosity twins. We compared 6 possible logistic models based on age, sex and three zygosity questions, to find the most discriminative model (Table 2). Single question about confused by strangers had a higher consistency rate than previously perceived zygosity and a complex strategy (Fig 1). Moreover, multivariable models had higher consistency rate than single variable models. The least prediction error from leave one out cross validation was 13.47%, suggesting that 86.53% of twins would be correctly classified in model 6.

**Table 1:** Characteristics of the 192 same sex twin pairs who were analyzed for questionnaire based zygosity determination.

		Monozygotic twin pairs (n = 125)		Dizygotic twin pairs (n = 67)		*P value* ^*a*^
		n	%	n	%	
**Age (years)**	**≤29**	17	10.5	11	16.4	0.2152
	**30–39**	24	14.8	18	26.9	
	**40–49**	50	30.9	16	23.9	
	**50–59**	19	11.7	10	14.9	
	**≥60**	15	9.3	12	17.9	
**Gender**	**Male**	83	51.2	48	71.6	0.4571
**Confused by strangers**	**Yes**	101	62.3	12	17.9	<0.0001
	**No**	21	13.0	53	79.1	
	**Hard to say**	3	1.9	2	3.0	
**Formal zygosity test**	**Yes**	20	12.3	3	4.5	0.00997
	**No**	62	38.3	47	70.1	
	**Do not know**	43	26.5	17	25.4	
**Previously perceived zygosity**	**MZ**	44	27.2	5	7.5	0.00014
	**DZ**	41	25.3	30	44.8	
	**Do not know**	40	24.7	32	47.8	

MZ: Monozygotic, DZ: Dizygotic, P-values were calculated from χ^2^ test for categorical variables

**Table 2:** Logistic models among 192 same sex twin pairs of questionnaire based information in predicting DNA-determined zygosity.

Items ^a^	Logistic Model ^b^	Prediction Accuracy ^c^				Cross Validation
	ln[p/(1–p)]	Cut-off point	Consistency rate	Sensitivity	Specificity	Prediction Error ^d^
**Model1: Previously perceived zygosity (PPZ)**	-0.22314–**1.95161I**_**PPZ1**_-0.08923I_PPZ2_	0.2623	0.5521	0.9254	0.3520	0.2124
**Model2: Confused by strangers (CBS)**	-0.4055–**1.7247I**_**S1**_+1.3312I_S2_	0.2531	0.8125	0.8290	0.8080	0.1471
**Model3: Formal zygosity test-PPZ-CBS**	-0.4055–**1.6740I**_**F1**_+0.8681I_F2_	0.2556	0.7500	0.8358	0.7040	0.1729
**Model4: PPZ+CBS**	+0.2256–**1.8418I**_**PPZ1**_-0.6647I_PPZ2_ **-1.7459I**_**S1**_+1.2943I_S2_	0.2998	0.8229	0.8209	0.8240	0.1403
**Model5: PPZ+CBS+Age**	-0.8366–**1.9203I**_**PPZ1**_-0.4749I_PPZ2_ **-1.9913I**_**S1**_+1.4618I_S2_ **+1.7361I**_**Age1**_+0.5644I_Age2_+1.0136I_Age3_ **+2.0168I**_**Age4**_	0.4838	0.8490	0.8209	0.8640	0.1354
**Model6: PPZ+CBS+Age+Gender**	-1.0594–**1.8973I**_**PPZ1**_-0.5284I_PPZ2_ **-1.9886I**_**S1**_+1.5088I_S2_+**1.5873I**_**Age1**_+0.4255I_Age2_+0.9723I_Age3_+**1.8744I**_**Age4**_+0.4884I_G_	0.5190	0.8698	0.8060	0.9040	0.1347

a. PPZ was the question about previously perceived zygosity, CBS was the question whether strangers confused by twins’ appearance, Formal zygosity test-PPZ-CBS was a complex strategy for zygosity determination (Fig 1).

b. p would be the probability of dizygotic (DZ) twins. I_PPZ1_, I_PPZ2,_ I_S1,_ I_S2,_ I_F1,_ I_F1_ referred to the dummy variables of question PPZ, CBS and Formal zygosity test-PPZ-CBS respectively, a subscript “1” indicated monozygotic (MZ) twins versus Do not know\Hard to say, subscript “2” indicated DZ versus Do not know\Hard to say. I_Age1-Age4_ were the dummy variables of age group 30–39, 40–49, 50–59 and ≥60 versus ≤29. I_G_ was the dummy variable of Male versus Female. Bolds represented *P-value* <0.05.

c. Cut-off point would be the average point of probability (of DZ) maximizing both sensitivity and specificity. Consistency rate would be the proportion of correctly diagnosed MZ and DZ pairs by the logistic model among 192 pairs.

d. Prediction Errors were the raw estimates calculated by the Leave-One-Out Cross Validation (LOOCV) method.

**Fig 3:**
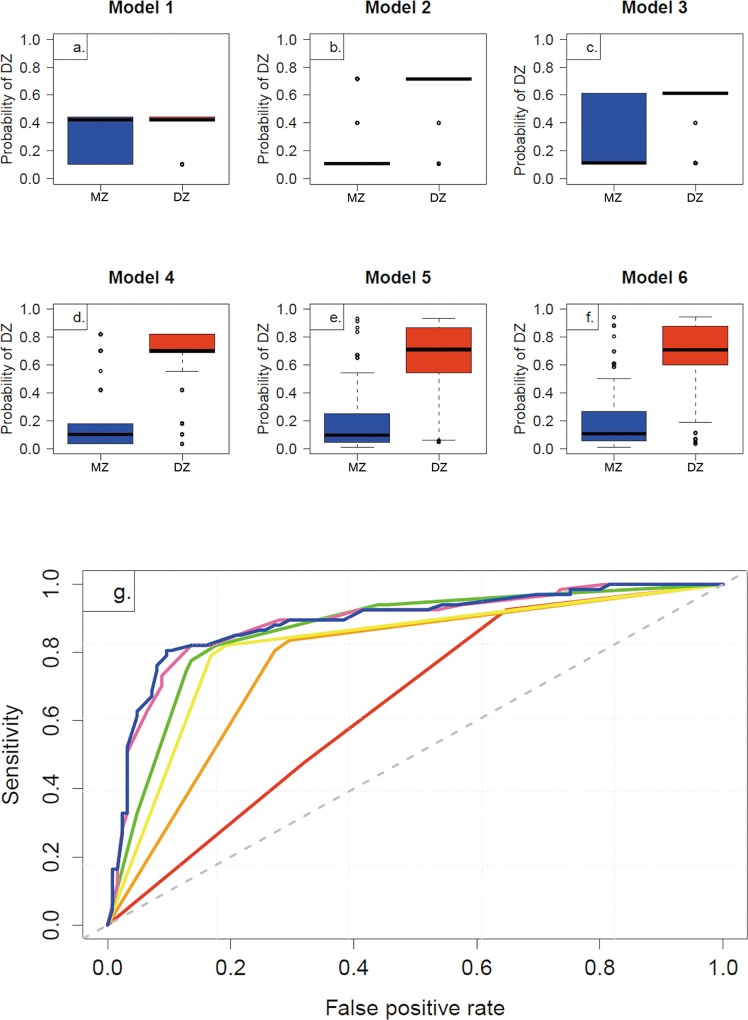
The discriminative ability compared among six logistic regression models by questionnaire zygosity determination. (a-f) Boxplot of probabilities for Dizygotic Twins (DZ) from the Monozygotic Twins (MZ) versus DZ logistic regression Model 1–6, and (g) ROC curves showing the discriminative ability of the MZ versus DZ under logistic regression model. Model 1–6 were denoted by a red, yellow, orange, green, pink and blue line, respectively. The Areas Under the Curve (AUC) and its 95% Confidence Interval was 64.54% (57.32%-71.76%), 82.14% (76.34%-87.95%), 77.55% (71.47%-83.64%), 86.49% (81.07%-91.91%), 88.67% (83.50%-93.85%) and 89.03% (83.86%-94.19%) for Model 1–6.

Fig 3A–3F showed boxplots of the fitted probabilities for DZ from six logistic regression models between MZ and DZ twins. The majority of MZ in model 2 had lower predicted probabilities for DZ, while the majority of DZ twins had higher predicted probabilities. Similar results were also found in model 4 and 6. But in model 1 and 3, we could not identify the majority of MZ would had lower predicted probabilities. The largest AUC (89.03%) model included variables of sex, age, confused by strangers and previously perceived zygosity (Fig 3G). The best cut-off point probability would be 0.5190, above which indicated to be DZ twins.

## Discussion

In this study, we used 65 SNPs in the 450k methylation array to determine zygosity and we found that the results of 65 SNPs were consistent with genotyping. In the questionnaire-based zygosity determination analyses, age, gender, questions of appearance confused by strangers and previously perceived zygosity consisted of the most predictable model with an AUC of 89.03% (95% CI: 83.86%-94.19%). For twin studies with genotyping and\or 450k methylation array, there would be no need to conduct STRs for the sake of costs consideration.

The 65 SNPs in the 450k methylation array could discriminate MZ from DZ twins, but the ability for discriminate DZ from other related individuals such as parents-offspring, half-siblings, cousins or unrelated ones was limited. As the results showed that DZ twins proportion of IBD ranged from 0.2064 to 1, we could not tell if there are any singletons or other related ones involved in the studies from methylation chips [2]. Some forensic studies have compared SNPs versus STRs in relationship analysis. SNPs were prone to have a better work on DNA amplification, lower mutation rates and less costs than STRs. On the contrary, STRs tended to have a better discriminative power and mixture samples identification than SNPs [25–27]. Hepler compared the use of 50 SNPs and 50 microsatellite loci on 15 unrelated and 15 parent–child pairs and found that one of the unrelated pairs had SNPs-based estimates that were close to the values found for half-siblings. It seemed that 50 SNPs were insufficient and that 200 SNPs or more would be needed to characterize relatedness [28]. Hannelius et al. studied 99 Caucasian twin pairs to compare a panel of 50 SNPs with 16 STRs, 2 pairs were assigned a different zygosity. Their SNPs-based method has a false positive rate less than 0.02, which might be acceptable in zygosity determination [3].

Questionnaires with multiple questions plus age and gender would be better performed than a single question about zygosity determination. The major question about zygosity was based on twins’ current facial appearance, which might be varied across age or gender [29–31]. Our results showed that the probability of DZ was positively related to age. It was possible that the accumulation of environmental difference between twins made them less alike [17]. The best model of questionnaire in zygosity determination had a consistency rate of 86.98% and an AUC of 89.03%. Most of other twin studies had a consistency rate more than 90% [16–18, 32, 33]. The only three questions about zygosity might not be sufficient, other information collected from twins’ parents, or teachers, or some physical resemblance were related to zygosity determination as well. Some anthropometric indicators might also be factors related to zygosity misperception. For example, because height and obesity influenced the overall appearance of the body, it was logical to indicate that some DZ twins share many physical characteristics, which made them appear as MZ twins. Similarly, a smaller proportion of MZ twins had discordant height or obesity, which made twins believe they do not look like alike, as did DZ twins [16].

The genotyping and 450k methylation array might bring benefits for both twin studies in developed and developing countries. For twin studies in developed countries, zygosity was based on questionnaire and DNA testing [34–38]; researchers could conduct methylation or genotyping experiments for those who were lacking of DNA zygosity testing. And for those who had DNA zygosity testing, the result of methylation might be a replication for previous tests. There were 13.1 twin births per 1000 births among 76 developing countries every year [39]. The value of twins in population-based studies has not been fully discovered. The twins in developing countries were usually newly recruited. Genotyping and methylation array could help researchers in developing countries to catch up with the latest trend of twin studies without costs in zygosity testing, and saving the amount of DNA samples.

There were two limitations in this study. Firstly, the zygosity questionnaire was lack of the external validation samples that might not be successfully applied in other samples or populations. The second one was that the answers of “Do not know” in question of formal zygosity test and previous perceived zygosity were relatively high in current study. Probably because the majority of twins were recruited in rural areas of China, they might not be aware of the questions due to the low education level in those areas.

In conclusion, 450k methylation array can be used in zygosity determination. Questionnaire-based zygosity assessment in Chinese adult twins can be regarded as a valid method in zygosity determination.

## Supporting Information

S1 Table Annotation profile of the 65 SNPs in 450k Methylation Array (EXCEL). (XLS)

S2 Table Zygosity Assignments by Relatedness Analysis through Genotyping and Methylation Array (EXCEL). (XLS)
